# Organizing pneumonia following ocrelizumab use in a patient with multiple sclerosis: A case report

**DOI:** 10.5578/tt.20239913

**Published:** 2023-03-10

**Authors:** N. AYSAN, G. KÖYBAŞI, C. SATICI, M.A. DEMİRKOL, B. A. YİĞİTBAŞ, A.F.A. KOŞAR

**Affiliations:** 1 Clinic of Chest Diseases, İstanbul Yedikule Chest Diseases and Thoracic Surgery Training and Research Hospital, İstanbul, Türkiye; 2 Clinic of Chest Diseases, Başakşehir Çam and Sakura City Hospital, İstanbul, Türkiye; 3 Department of Chest Diseases, İstanbul Medeniyet University Faculty of Medicine, İstanbul, Türkiye

**Keywords:** organizing pneumonia, drug induced organizing pneumonia

## Abstract

Organizing pneumonia following ocrelizumab use in a patient with multiple sclerosis: A case report.

Ocrelizumab is an anti-CD20 monoclonal antibody used in the treatment of primary progressive and relapsing multiple sclerosis (MS). Although cases of organizing pneumonia have been reported in association with other antiCD20 agents such as rituximab, there is insufficient data in the literature on Ocrelizumab-associated lung involvement. Herein, we present a case of organizing pneumonia in a 37-year-old female patient with multiple sclerosis following Ocrelizumab use.

## Abstract (tr)

Multipl Skleroz tanılı bir hastada ocrelizumab kullanımını takip eden organize pnömoni : Vaka sunumu

Ocrelizumab, primer progresif ve tekrarlayan multipl skleroz (MS) tedavisinde kullanılan bir anti-CD20 monoklonal antikorudur. Rituximab gibi diğer anti-CD20 ajanları ile ilişkili organize pnömoni vakaları olduğu bilinse de Ocrelizumab ilişkili akciğer tutulumu ile ilgili literatürde yeterince veri bulunmamaktadır. Bu olgu sunumumuzda, MS tanısı nedeniyle Ocrelizumab kullanan 37 yaşındaki kadın hastada, Ocrelizumab kullanımı sonrası gelişen organize pnömoni olgusunu sunmaktayız.

## Introduction

Ocrelizumab, a humanized anti-CD-20 monoclonal antibody, has been used for both relapsing and primary progressive forms of multiple sclerosis ( 1 ). Anti-CD-20 agents such as rituximab are commonly associated with adverse reactions, including infusion-related reactions and influenza-like symptoms ( 2 ). Apart from these minor side effects, major adverse effects such as organizing pneumonia and ARDS are also documented, which are extremely rare but can be fatal ( 3 , 4 ). Several cases of rituximab-related lung toxicity have been reported. However, there is a lack of data regarding the effect of a relatively new anti-CD-20 agent ocrelizumab on the respiratory system ( 5 , 6 ). Herein, we present a case of organizing pneumonia related to ocrelizumab use, which is the first reported case to the best of our knowledge.

## CASE REPORT

**Figure 1:**
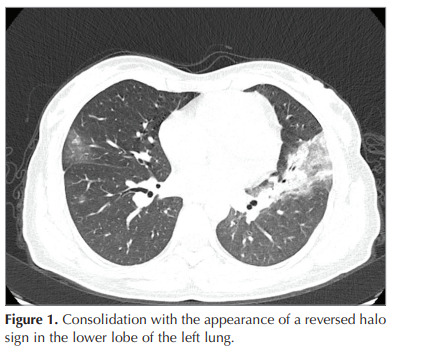
Consolidation with the appearance of a reversed halo sign in the lower lobe of the left lung.

**Figure 2:**
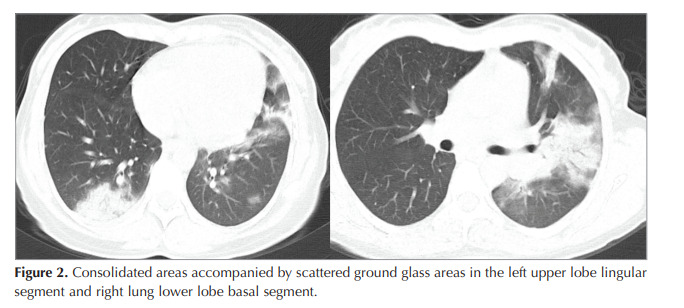
Consolidated areas accompanied by scattered ground glass areas in the left upper lobe lingular segment and right lung lower lobe basal segment.

**Figure 3:**
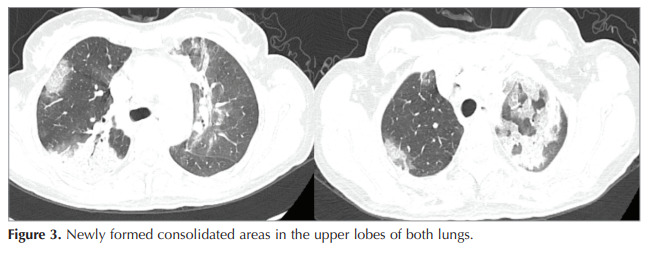
Analysis of MUC5B and TERT genes among study groups.

A 37-year-old female presented with a dry cough and intermittent fever that had persisted for two months, and a gradual increase in shortness of breath. She was diagnosed with multiple sclerosis and had been using fingolimod treatment for seven years. Since the disease had a progressive course, ocrelizumab treatment (300 mg/day) was started three months ago. This treatment was administered to the patient as 300 mg twice a day, with an interval of two weeks, and the next dose was not administered. She indicated that her symptoms had started after the onset of ocrelizumab treatment. She had no chronic diseases and was not on any other medication. She had no history of smoking, and her vital signs were normal except for the body temperature of 38.3°C. A pulmonary examination showed clear breath sounds. There were no abnormal physical examination findings in the other systems. An initial laboratory work-up showed an increased C-reactive protein level of 60 mg/L. A chest X-ray showed lobar pneumonia and a consolidated area with a reversed halo appearance detected in the thorax computed tomography (CT). Oral moxifloxacin therapy was started (Figure 1). One week after the onset of the treatment, the patient was hospitalized due to an acute worsening of dyspnea and high fever. The CT scan demonstrated multiple consolidated areas accompanied by scattered ground glass opacities bilaterally, predominantly in the lingular segment of the left upper lobe and the basal segment of the right lower lobe (Figure 2). Blood and sputum cultures were sterile and polymerase chain reaction tests for viral infections, including COVID-19, were found to be negative. Serological tests for connective tissue disease were also normal, including anti-nuclear antibodies, anti-neutrophil cytoplasmic antibodies, and rheumatoid factor. Two weeks after oral moxifloxacin therapy, there was no clinical improvement, and a

chest X-ray revealed that consolidation in the lower lobe of the left lung had disappeared, and new consolidated areas in the upper lobes of both lungs were observed (Figure 3). Bronchoalveolar lavage was performed in the middle lobe of the right lung, and cytological analysis showed alveolar histiocytes, lymphocytes, and epithelial cells. Bronchial lavage cultures were found to be sterile. With the preliminary diagnosis of organizing pneumonia, she was started on 32 mg methylprednisolone and her complaints of fever and dyspnea improved dramatically in 14 days.

**Figure 4:**
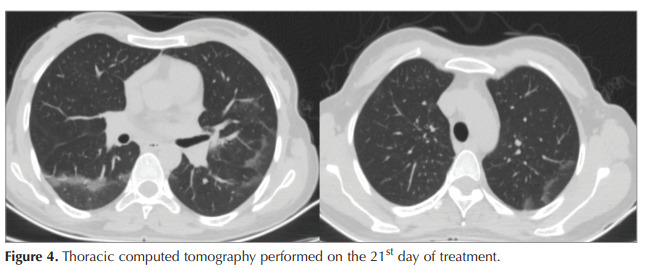
Thoracic computed tomography performed on the 21st day of treatment.

**Figure 5:**
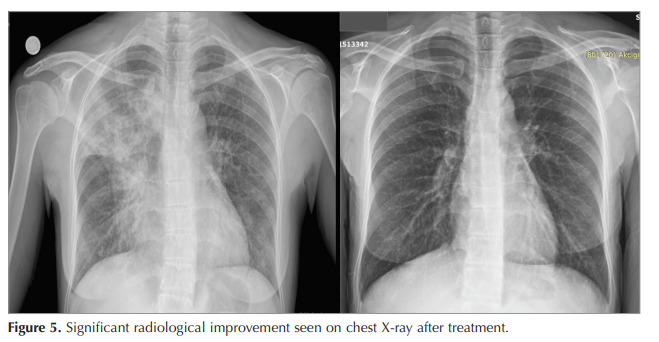
Significant radiological improvement seen on chest X-ray after treatment.

In line with these findings, the patient was diagnosed with ocrelizumab-induced organizing pneumonia, and she was discharged on methylprednisolone treatment. A follow-up thorax CT scan on the 21st day of the treatment revealed that the consolidated areas were significantly resolved (Figure 4). During the follow-up, the patient continued to receive methylprednisolone treatment, which was tapered off after three months. Significant clinical and radiological improvement was observed (Figure 5). As the patient was diagnosed with ocrelizumab-related organizing

pneumonia, ocrelizumab treatment was not restarted. The patient is still being monitored, and no evidence of recurrence has been found in the past year.

### Di

Organizing pneumonia is a form of diffuse interstitial lung disease characterized by a granulomatous pattern of fibroblasts and myofibroblasts accumulating in a distal intra-alveolar space. The disease is most commonly idiopathic, but some of the known causes of organizing pneumonia include radiation therapy, infection, drugs, connective tissue disorders, or immunosuppression ( 7 ). The most common symptoms (95%) are cough, fever, malaise, and shortness of breath ( 8 ). The physical examination of patients with organizing pneumonia usually reveals inspiratory crackles. Since organizing pneumonia is linked to connective tissue diseases, extrapulmonary symptoms such as muscular weakness, sclerodactyly, or skin lesions might be detected. Laboratory findings are nonspecific, but increased CRP or ESR levels are common. Although the pulmonary function test is mostly normal, a restrictive pattern can also be detected ( 9 ).

Bilateral alveolar infiltrates are the most common radiological findings on chest X-rays. Occasionally, migratory transient peripheral alveolar infiltrates can be seen. Computed tomography reveals patchy alveolar infiltrates in the form of ground glass opacities involving the peripheral and lower zones. This might be followed by nodular appearance, pleural effusion, and septal thickening ( 10 ).

In our case, the major symptoms were dyspnea and intermittent fever accompanied by increased CRP levels. A lack of response to empiric antibiotics for community-acquired pneumonia was the main clue for further examinations. The radiological appearance was typical for organizing pneumonia with a peripheral distribution of consolidations, ground glass opacities, and migratory infiltrates. Even though these findings are not pathognomonic, they are strongly indicative of organizing pneumonia when combined with the patient’s history and clinical setting ( 11 ).

Although there is no consensus on the mechanisms of drug-induced interstitial lung diseases, oxidative damage, pulmonary vascular damage, and damage due to phospholipid accumulation may be responsible. The most common drugs causing organizing pneumonia are amiodarone, amphotericin, bleomycin, cyclophosphamide, interferon beta, nitrofurantoin, and penicillamine ( 12 ). Our patient was using ocrelizumab and symptoms occurred right after the treatment. Ocrelizumab is a monoclonal antibody against the CD-20 antigen and is approved for the treatment of multiple sclerosis. The common adverse events are infusion-related reactions, nasopharyngitis, upper respiratory tract infections, headaches, and urinary tract infections. Although there is a lack of data about the lung toxicity of ocrelizumab, cases of organizing pneumonia due to another anti-CD-20 agent, rituximab, have been reported ( 3 , 5 , 6 ). Even though rituximab has been used for the treatment of connective tissue diseaserelated interstitial lung diseases with promising outcomes, fatal lung toxicity has also been reported ( 4 ). There were no occurrences of organizing pneumonia among the pulmonary side effects of ocrelizumab in the literature, although there have been cases of rituximab-related organizing pneumonia.

Corticosteroids are frequently used for the treatment of organizing pneumonia. The general approach is to initially administer a proper dose of corticosteroids based on the severity of the lung involvement (0.5- 1.5 mg/kg per day of prednisolone) for 4–8 weeks followed by a lower dose for 4-6 weeks. Clinical improvement begins in a few days, yet radiological improvement might take a few weeks. Although relapses may occur after dose reduction or discontinuation of the corticosteroid therapy, retreatment usually responds. In order to avoid relapses, it’s also crucial to identify the etiology of the disease. In the case of drug-associated organizing pneumonia, the first step of treatment should be discontinuation of the drug ( 3 , 13 ). Response to corticosteroid treatment is excellent in the vast majority of patients. While most patients require treatment for six to 12 months, some may require extended treatment due to relapses ( 14 ).

### CONCLUSION

To the best of our knowledge, this is the first case report of organizing pneumonia following ocrelizumab use. In light of this example, we recommend close monitoring of patients on ocrelizumab for the onset of new respiratory symptoms and investigating whether lung involvement occurs early on.

#### CONFLICT of INTEREST

The authors have no conflict of interest to declare.

### AUTHORSHIP CONTRIBUTIONS

Concept/Design: NA, BAY, FK

Analysis/Interpretation: NA, GK, MAD

Data acqusition: NA, GK, MAD

Writing: NA, GK, CS, MAD

Clinical Revision: FK, CS, BAY

Final Approval: FK, CS, BAY
